# Independent component and pathway-based analysis of miRNA-regulated gene expression in a model of type 1 diabetes

**DOI:** 10.1186/1471-2164-12-97

**Published:** 2011-02-04

**Authors:** Claus H Bang-Berthelsen, Lykke Pedersen, Tina Fløyel, Peter H Hagedorn, Titus Gylvin, Flemming Pociot

**Affiliations:** 1 Glostrup Research Institute, Glostrup University Hospital, DK-2600 Glostrup, Denmark; 2 Hagedorn Research Institute, Niels Steensensvej 6, DK-2820 Gentofte, Denmark; 3 Center for Models of Life, University of Copenhagen, Blegdamsvej 17, DK-2100 Copenhagen, Denmark; 4 Center for Biological Sequence Analysis, Department of Systems Biology, Technical University of Denmark, DK-2800 Lyngby, Denmark; 5 Steno Diabetes Center, Niels Steensensvej 2, DK-2820 Gentofte, Denmark; 6 University of Lund, CRC, Skåne University Hospital, SE-20502 Malmoe, Sweden; 7 Department of Molecular Biomedicine, LEO Pharma A/S, Industriparken 55, DK-2750 Ballerup, Denmark

## Abstract

**Background:**

Several approaches have been developed for miRNA target prediction, including methods that incorporate expression profiling. However the methods are still in need of improvements due to a high false discovery rate. So far, none of the methods have used independent component analysis (ICA). Here, we developed a novel target prediction method based on ICA that incorporates both seed matching and expression profiling of miRNA and mRNA expressions. The method was applied on a cellular model of type 1 diabetes.

**Results:**

Microrray profiling identified eight miRNAs (miR-124/128/192/194/204/375/672/708) with differential expression. Applying ICA on the mRNA profiling data revealed five significant independent components (ICs) correlating to the experimental conditions. The five ICs also captured the miRNA expressions by explaining >97% of their variance. By using ICA, seven of the eight miRNAs showed significant enrichment of sequence predicted targets, compared to only four miRNAs when using simple negative correlation. The ICs were enriched for miRNA targets that function in diabetes-relevant pathways e.g. type 1 and type 2 diabetes and maturity onset diabetes of the young (MODY).

**Conclusions:**

In this study, ICA was applied as an attempt to separate the various factors that influence the mRNA expression in order to identify miRNA targets. The results suggest that ICA is better at identifying miRNA targets than negative correlation. Additionally, combining ICA and pathway analysis constitutes a means for prioritizing between the predicted miRNA targets. Applying the method on a model of type 1 diabetes resulted in identification of eight miRNAs that appear to affect pathways of relevance to disease mechanisms in diabetes.

## Background

microRNAs (miRNAs) are a class of small non-coding RNAs that function as posttranscriptional regulators of gene expression by mediating translational inhibition or mRNA degradation. miRNA bind to "seed" sites, i.e. stretches of 6-8 nucleotides in the 3' untranslated region (UTR) of their target mRNAs. miRNAs regulate various cellular processes and appear to be involved in the development of many diseases.

Most approaches for miRNA target identification rely on either one or a combination of seed matching, site accessibility and phylogenetic conservation [1]. In addition, some have incorporated target site location, multiple target sites and expression profiling [1]. A number of target prediction methods use expression profiling of both miRNA and mRNA expressions [2-5]. Most of the approaches are based on negative correlation, i.e. reciprocal expressions of miRNAs and their degraded target mRNAs.

High-dimensional biological data, such as microarray profiling data, are often interpreted as being composed of sets of transcriptional- or activity programs that explain some, or most, of the complexity in the data [6-8]. Various methods are being applied on profiling data e.g. principal component analysis (PCA) and clustering. In the last couple of years independent component analysis (ICA) has been extensively applied on mRNA profiling data and recently, ICA was applied on miRNA profiling data as well [9].

ICA is a computational method for separating mixed independent signals and can be used to decompose the expression matrix into independent components [10]. This decomposition has been shown to be informative in several studies [11-15], and superior to clustering and PCA [16-19]. Apparently, the representation of gene expression as a mix of independent, possibly overlapping, transcriptional programs captures the differential regulation of well-defined biological processes and metabolic pathways [19,20].

Type 1 diabetes (T1D) is an immune-mediated disease characterized by insulin deficiency due to a specific destruction of the pancreatic β-cells. Pro-inflammatory cytokines are involved in the destruction through induction of apoptosis [21]. The endocrine cells in the pancreatic islets all arise from the same progenitor stem cell and the maturation of the different cell types is dependent on a number of factors, such as transcription factors and miRNAs, that are activated in a tightly regulated pattern [22,23]. One of the central transcription factors in pancreatic development is Pancreatic and duodenal homeobox 1 (Pdx-1) [24-26]. In the mature endocrine cells, Pdx-1 expression is restricted to the β-cell, where it is important for insulin expression [27].

In the present study, we have used a model of type 1 diabetes based on β-cell maturation and interleukin-1β (IL-1β) sensitivity. In response to Pdx-1 induction the cells progress from an immature to a mature β-cell phenotype [28]. The β-cell maturation is accompanied by an increased sensitivity to the toxic effects of IL-1β that is reflected in both transcriptional and protein expression patterns [29-31]. Genes regulated by Pdx-1 are therefore believed to be involved in the acquired IL-1β sensitivity, and identification of these genes would provide knowledge about the mechanisms underlying this β-cell specific trait. Interestingly, a study investigating genomic targets of transcription factors in a β-cell line suggested that several miRNAs are under Pdx-1 regulation [32]. Furthermore, a number of miRNAs have been implicated in the regulation of pancreatic development and β-cell differentiation [22,33-36].

Here, we have developed a novel miRNA target prediction method that is based on ICA and combines seed matching and expression profiling. We comprehensively profiled both miRNA and mRNA expressions from the type 1 diabetes model. ICA was applied on the mRNA expression data for identification of miRNA targets. Our method was compared to negative correlation. We validated our observations by use of pathway analysis and human pancreatic islet preparations.

## Results

### β-cell specific gene expression

To confirm that the INSrαβ cells progressed from a hybrid αβ-like phenotype to a more mature β-cell phenotype upon Pdx-1 induction as seen in previous studies [28,37], the expression levels of known insulin and Pdx-1 dependent genes were examined using the array data (Additional file 1). The observed expression profiles were in agreement with insulin and Pdx-1 regulations known from other studies.

**Figure 1:**
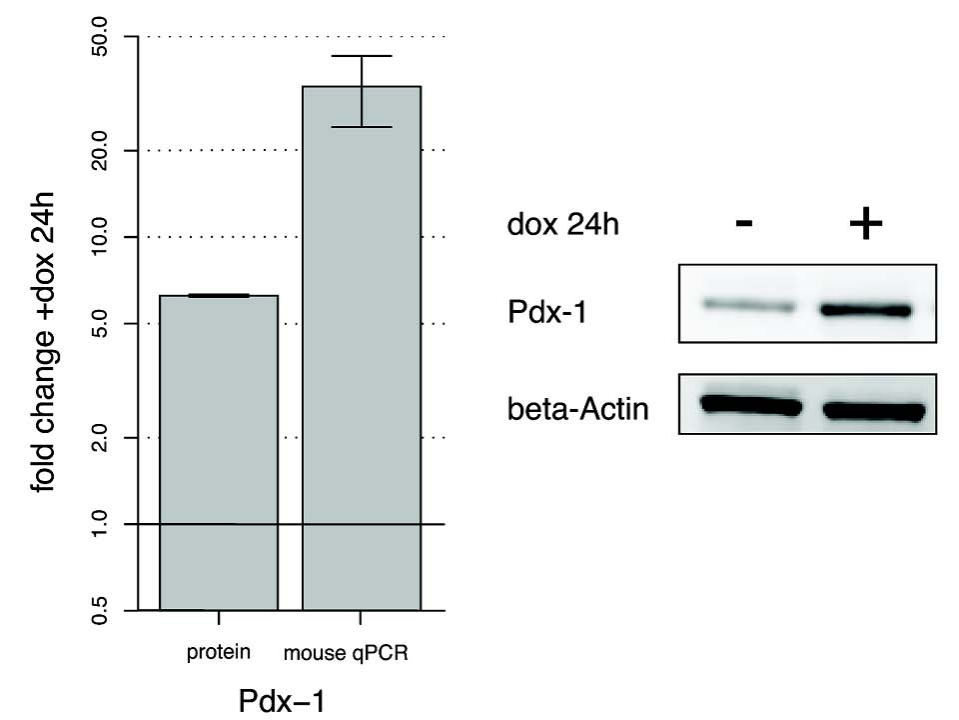
**Pdx-1 induction in the INSrαβ cell line**. Fold changes (mean and standard deviations) in Pdx-1 protein and mRNA after treatment with doxycycline (dox) for 24 h. The gels to the right represent Pdx-1 and β-actin induction with and without dox stimulation for 24 h.

The dox-induced Pdx-1 expression was examined using qPCR and western blotting with mouse-specific primers and antibody, respectively. We found that dox treatment for 24 hours resulted in increased expression of both Pdx-1 mRNA (33 fold, p < 0.05) and protein (6 fold, p < 0.05) (Figure 1).

### miRNAs differentially expressed in a model of type 1 diabetes

**Figure 2:**
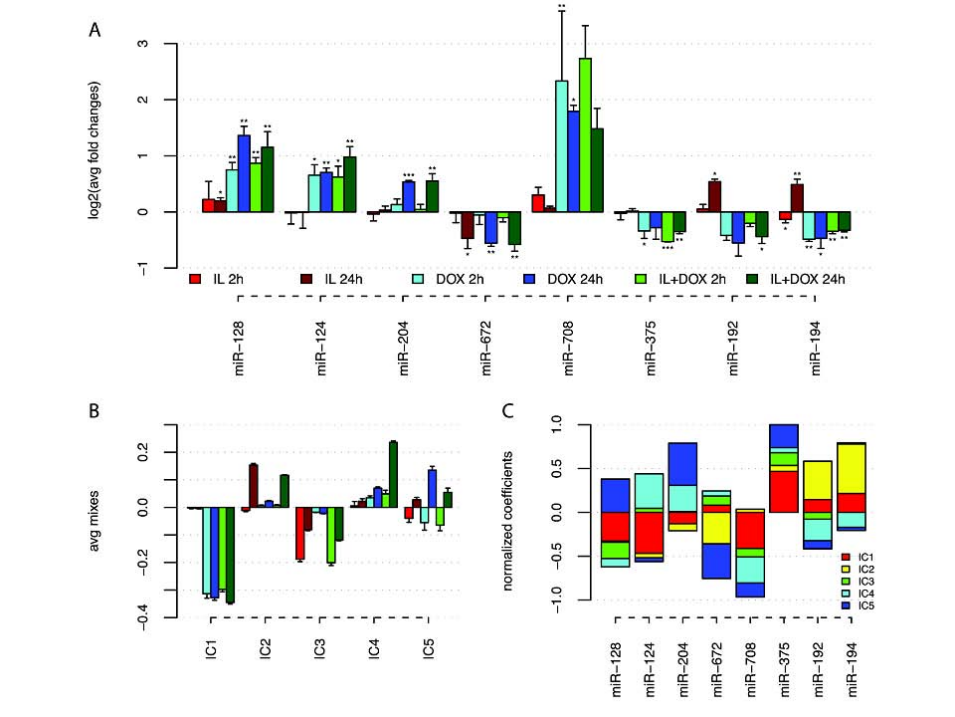
**miRNA expressions and mixes of independent components**. There are three experimental conditions: Pdx-1 induction (dox treatment), IL-1β treatment and time (samples are taken 2 h and 24 h after treatment). (A) Log2-transformed fold changes (mean and standard deviations) between experimental and control (untreated cells) conditions. *: 0.05 > q > 0.01, **: 0.01 > q > 0.001, ***: 0.001 > q > 0. (B) For each independent component (IC) the average of mixes are shown for each condition. Bars represent mean and standard deviation. IC1 is a Pdx-1 component showing mixes correlating to Pdx-1 induction. IC 2 and 3 are cytokine components with mixes correlating to IL-1β treatment. IC 4 shows mixes correlating to induction of Pdx-1 and treatment with IL-1β after 24 h. IC 5 has mixes increasing from 2 h to 24 h in all three conditions. (C) The coefficient for the linear combination of the ICs giving the best fit of the miRNA expressions. The coefficients are scaled to have an absolute sum of one.

miRNA expression profiling resulted in identification of eight miRNAs with differential expression in response to Pdx-1 induction and/or IL-1β exposure. All eight miRNAs (miR-124/128/192/194/204/375/672/708) showed significant (p < 0.05) changes in expression between conditions and/or time points (Figure 2A). The eight miRNA expression profiles capture all three experimental conditions: dox-induced Pdx-1 expression, IL-1β exposure and time. For example, the miR-672 expression decreased significantly (p < 0.05) with time independent of Pdx-1 induction and/or IL-1β treatment. The reversed pattern was seen for miR-204, though only for cells with induced Pdx-1 expression. A third example was the up-regulating effect of IL-1β treatment on the expressions of miR-128/192/194.

**Figure 3:**
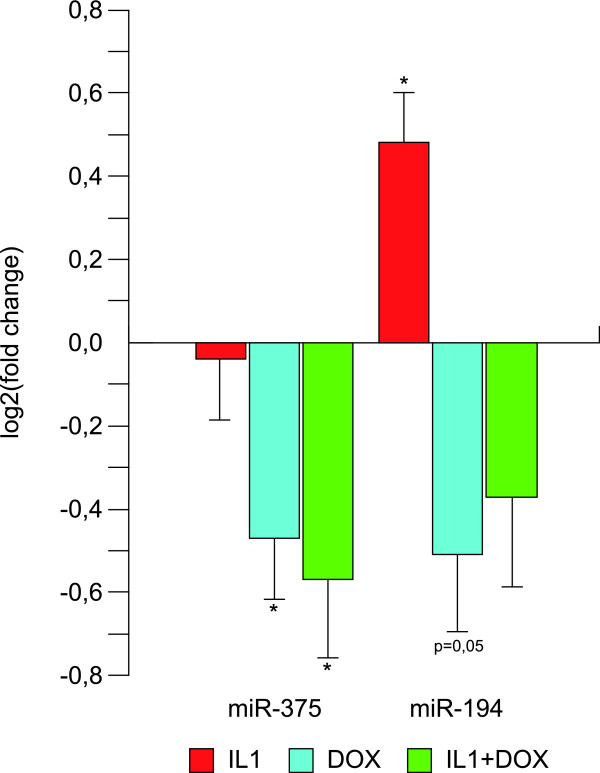
**Expression of miR-375 and miR-194 using real-time qPCR**. Log2-transformed fold changes (mean and standard deviation) between experimental and control condition after Pdx-1 induction (dox) and/or IL-1β stimulation for 24 h. *: p < 0.05.

The expression of two of the eight miRNAs were validated in a new set of samples using real-time quantitative PCR. We found that the expression of miR-375 was significantly decreased in response to Pdx-1 induction (by ~50%, p < 0.05), whereas the miR-194 expression was significantly increased in response to IL-1β treatment (by ~50%, p < 0.05), see Figure 3. Furthermore, there was a tendency towards a decreased miR-194 expression in response to Pdx-1 induction (by ~50%, p = 0.05). These expression changes confirmed the results from the array.

### miRNA target prediction using negative correlation or ICA

To assess the effect of the Pdx-1- and IL-1β-mediated miRNA activity, mRNAs were profiled in the same set of samples as used for the miRNA arrays.

Initially, targets were predicted for each of the eight miRNAs using 6mer seed matching. The total number of 6mer seed matches for each miRNA is listed in Table 1. For identification of miRNA targets using negative correlation, correlation coefficients were calculated between each pair of miRNA and mRNA profiles and the mRNAs were ordered according to these correlation coefficients. Four of the eight miRNAs showed a significant enrichment of sequence predicted targets among mRNAs with correlation coefficients close to -1 (q < 0.1), Table 1. Additional file 2 lists all calculated correlation coefficients.

**Table 1:** Target prediction using negative correlation or ICA (loads)

miRNA	# 6mer matches	Correlation (q-value)	Load (q-value)
miR-124	2215	0.0027	0.005

miR-128	2105	0.63	0.07

miR-192	1128	0.63	0.0007

miR-194	1110	0.0016	0.02

miR-204	1560	0.63	0.02

miR-375	1128	0.0027	0.04

miR-672	1060	0.87	0.5

miR-708	1675	0.023	0.0007

The table shows the number of 6mer seed matches in the array data for each of the eight miRNAs (2^nd ^column), the q-values (corrected p-values) for negative correlation (3^rd ^column) or load values (4^th ^column). The q-values in the 4^th ^column are the lowest q-value found among the five significant ICs.

Applying ICA on the mRNA expression data resulted in identification of five highly significant ICs (Bonferroni corrected q < 0.0001) with mixes shown in Figure 2B. See Additional file 2 for all loads, as well as the method section for further details on ICA.

For each of the eight miRNAs, the loads in each IC were tested for enrichment of predicted targets with a 6mer seed match. Seven of the eight miRNAs (including all four identified by negative correlation) showed a significant enrichment of sequence predicted targets in the IC loads (q < 0.07), Table 1. Furthermore, we analyzed IC 1 for cooperativity between each pair of miRNAs by testing the loads in IC 1 for enrichment of predicted targets with seed matches for both miRNAs (Additional file 3).

We found that the miRNA pairs miR-375/672, miR-194/375, miR-192/375 and miR-124/194 had a significant regulatory effect (Bonferroni corrected q < 0.05) when testing for enrichment of their common targets in IC 1.

The miRNAs had small fold changes compared to the fold changes of the mRNAs. Therefore it was not possible to apply ICA on the miRNA data or on a combination of the mRNA and miRNA expression matrices. However, by forming a linear superposition of the ICs we identified the major contributions from the ICs on the miRNA expressions. Interestingly, all eight miRNAs could be represented (percent variance explained >97%) by a superposition of the five ICs, as shown in Figure 2C (also see Additional file 4). The majority of predicted miRNA targets were present in two or three of the ICs. For example, miR-124 primarily has targets in component 1 and 4, which both are Pdx-1 affected components. Another example is miR-192 and miR-194 that both have targets in component 1, 2 and 4, which were affected by both Pdx-1 and IL-1β.

### Independent components have clear biological profiles

To assess the biological relevance of the identified ICs, we tested the ICs for enrichment of genes known to be regulated by Pdx-1 or affected by IL-1β (Additional file 5). Mixes of IC 1 clearly correlated with the induction of Pdx-1, and genes regulated by Pdx-1 were overrepresented in this IC (p < 0.047, data not shown). Interestingly, one group had low negative loads and another had high positive loads, indicating positive and negative regulation by Pdx-1, respectively. Similarly, ICs 3 and 4 correlated with the presence or absence of IL-1β. Specifically, IC 3 was enriched for known IL-1β affected genes among genes with low negative load (p < 0.0021, data not shown), whereas IC 4 was enriched for IL-1β affected genes with high positive load (p < 0.024, data not shown).

In a similar manner, we tested the ICs for enrichment of miRNA targets associated with metabolic or signalling pathways, as annotated by KEGG [38] or by the Molecular Signatures Database (MSigDB, http://www.broadinstitute.org). Specifically, we tested the genes with high positive or low negative loads in each IC for overrepresentation of annotated pathway genes.

**Figure 4:**
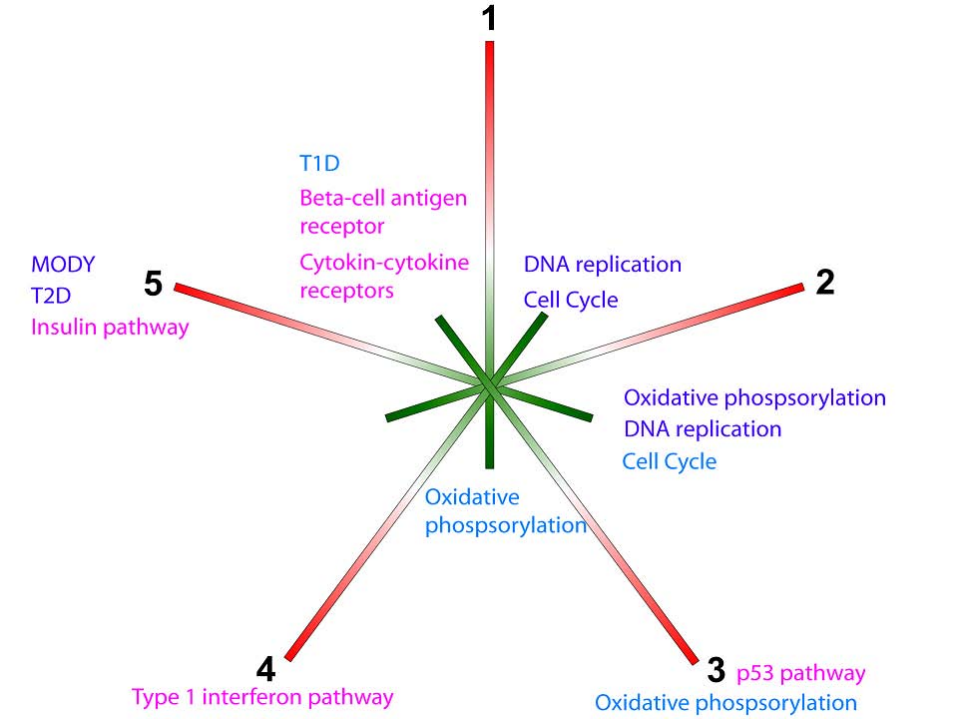
**miRNA target-enriched pathways**. Diagram showing a selection of the significant pathways for the miRNA targets in the five ICs when using KEGG annotations (blue text), MSigDB annotations (magenta text) or both annotations (purple text). The color of the bars indicate positive loads (red line) and negative loads (green line) of the miRNA targets in the ICs.

When using KEGG terms only, 25 pathways were significantly enriched for miRNA targets in the five ICs (q < 0.05). The most significant and diabetes-relevant pathways are shown in Figure 4 (all are listed in Additional file 6). Notable was the significance of genes with low loads in IC 3 belonging to the T1D pathway. IC 3 had a clear correlation with stimulation of IL-1β. Furthermore, the pathways maturity onset diabetes of the young (MODY), type 2 diabetes (T2D) mellitus and oxidative phosphorylation were significant. The first two have an obvious relation to diabetes, and the oxidative phosphorylation pathway has been shown to be related to both type 1 and type 2 diabetes [39]. When using MSigDB annotations, 150 pathways were significantly enriched for miRNA targets belonging to the five ICs (q < 0.05). A selection of the pathways is shown in Figure 4 (all are listed in Additional file 6). Since KEGG is part of MSigDB it comes as no surprise that T2D, MODY and oxidative phosphorylation again showed up as significant. However, T1D did not show up as a significant pathway, probably due to correction of multiple testing, since MSigDB is a much larger repository. Also, dysregulation of genes involved in the p53 signaling pathway have been suggested to sensitize the cells to apoptotic stimuli [32,40]. In accordance with this, we find genes annotated with the p53-signalling pathway (using MSigDB) having significant low loads in IC 3.

### Identification of miRNA regulatory networks

miRNAs and mRNAs can interact in regulatory networks. miRNAs can regulate mRNAs directly or indirectly through secondary factors. Furthermore, mRNA targets can act as transcription factors for miRNAs, thus forming a regulatory loop.

**Figure 5:**
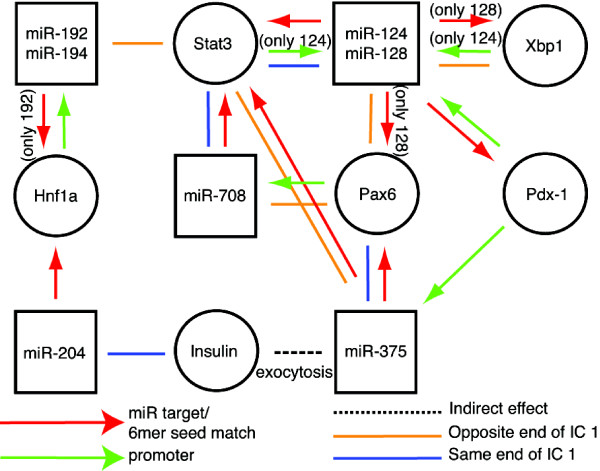
**miRNA regulatory network**. miRNA regulatory networks were identified using a combined bioinformatics approach involving seed matching, promoter analysis, text mining and ICA. The nodes and links were established by linking miRNAs with potential mRNA targets or transcription factors.

Using a combined bioinformatics approach involving seed matching, promoter analysis, text mining and ICA we identified miRNA regulatory networks (Figure 5). Using the miRNA database http://www.miRbase.org, we found STAT3 binding sites in the promoter of the human miR-124 gene. This interaction was supported by ICA that showed that miR-124 and Stat3 both have negative contribution from IC 1 (Additional file 2 and Figure 2C) correlating with an up-regulation in response to Pdx-1 induction. Interestingly, the 3'UTR of Stat3 has seed matches for miR-124 (TargetScan and 6mer seed matching), indicating a potential feedback loop.

Furthermore, we found positive and negative contribution from IC 1 for Pax6 and miR-124/128, respectively (Additional file 2 and Figure 2C), meaning they have reciprocal expressions (down-regulation of Pax6 and up-regulation of miR-124/128) in response to Pdx-1 induction. Interestingly, the 3'UTR of Pax6 contains a 6mer seed match for miR-128 indicating potential repression of Pax6 by miR-128.

### Validation in human pancreatic islets and β-cell studies

To verify the identified miRNA targets affected by Pdx-1 and/or cytokine stimulation, we used mRNA expression profiling data from studies on human pancreatic islets and rat β-cell lines and tested whether the data were enriched for ICA-identified targets of the eight miRNAs.

Using mRNA profiling data from untreated and cytokine treated human pancreatic islet preparations (unpublished data) we tested the cytokine-induced fold changes for enrichment of predicted miRNA targets. Interestingly, we found that predicted targets of miR-128/192/194/204/375 were significantly up-regulated in response to cytokine treatment as compared to non-targets (q = 0.007, q = 0.004, q = 0.033, q = 0.001 and q = 0.05, respectively). The results suggest that the five miRNAs may play a role in human pancreatic islets as well.

Additionally, we performed similar analysis of publicly available data from two previous studies on β-cells. Cardozo et al. [41] performed mRNA profiling on β-cells from 10 weeks old male Wistar rats, un-stimulated or stimulated with cytokines (IL-1β and/or IFNγ). Using their mRNA data, we found that targets of miR-192 were significantly down-regulated in response to IL-1β stimulation when comparing to non-targets (q = 0.02). Kutlu et al. [42] performed mRNA profiling on insulin producing INS-1E cells, untreated or exposed to a combination of IL-1β and IFNγ. In their data set, we found that the expressions of miR-128 targets were significant increased in response to cytokine exposure when comparing to non-targets (q = 0.002).

## Discussion

The motivation for applying a more advanced method like ICA for miRNA target prediction than negative correlation was that surprisingly few mRNAs have clear negative correlations with their targeting miRNAs. This is probably due to the mRNA profiles being influenced by a number of factors e.g. miRNA regulation, transcription factor binding and site accessibility. Here, ICA was used as an attempt to filter the factors influencing mRNA expression. Decomposition of microarray data using ICA has been shown to outperform other linear data representations, such as PCA [16-19]. Several target prediction methods have incorporated miRNA and mRNA profiling data. However, none of them uses ICA.

The miRNA array profiling identified eight miRNAs with differential expression in a type 1 diabetes model. Performing ICA on the mRNA expressions from the same samples resulted in five ICs that correlated with the experimental conditions studied. Comparing the two target prediction methods indicated that ICA was better at capturing miRNA activity than negative correlation. Seven miRNAs showed a significant enrichment of sequence-predicted targets when using ICA, as compared to only four by use of negative correlation. Interestingly, the ICs were enriched for miRNA targets with functional roles in diabetes-relevant pathways e.g. the pathways T1D, T2D, MODY, oxidative phosphorylation, insulin, cytokine-cytokine receptors and type 1 interferon. This supports that the eight miRNAs are implicated in disease mechanisms in diabetes. Additionally, targets of five of the eight miRNAs were significantly regulated by cytokines in models of β-cell destruction e.g. in human islets.

miRNAs fine tune the expression of genes in a combinatorial manner, meaning that several miRNAs can target the same mRNA transcript [43]. Furthermore, a cluster of co-expressed miRNAs can regulate functionally related genes [44]. In this study, we observe small expression changes in the miRNAs. However, even minute changes in miRNA expressions might have impact on mRNA expression, and miRNAs acting in a cooperative manner can most likely induce biologically relevant expression changes in their targets. ICA can uncover these more complex interactions. Interestingly, it was recently suggested that cooperativity could be incorporated for prediction of target interactions between different miRNAs [1]. For the eight miRNAs we identified, there is a significant overlap in the mRNAs they target. We have incorporated cooperativity between miRNAs pairwise and identified four miRNA pairs (miR-375/672, miR-194/375, miR-192/375, miR-124/194) that had a significant co-regulatory effect on their common targets in IC 1.

Of the eight significant miRNAs, miR-124 and miR-375 have previously been identified in β-cells [33,35,45,46]. Further, the expression of miR-204 has been shown to be induced in insulinomas, where its expression correlated with insulin expression [47].

That miR-375 was significantly regulated strengthens our model since a previous study observed interaction between Pdx-1 (and NeuroD1) and the miR-375 locus [32]. However, no Pdx-1 consensus binding sites were identified, but binding elements for other transcription factors have been identified in the miR-375 locus [48]. The decreased miR-375 expression could, at least in part, be mediated through NeuroD1, since we observed a decreased NeuroD1 expression in response to Pdx-1 induction. Additionally, the decreased miR-375 expression is in agreement with miR-375 having a higher expression level in non-β-cells than in β-cells [35]. This is also supported by our findings in α-cells versus β-cells (Additional file 7). Interestingly, both miR-375 and Pax6, a key factor in α-cell development, had negative loads in IC 1, i.e. both were down-regulated in response to Pdx-1. The decreased miR-375 expression is also in compliance with the function of miR-375 as a negative regulator of insulin exocytosis [45], since it correlates with the need for an increased insulin secretion in the mature β-cell phenotype. Similarly, miR-124 has been shown to modulate insulin secretion by targeting Foxa2 [33]. miR-194 is highly expressed in liver and in intestinal epithelial cells, where it is under regulation by Hnf1α [49,50]. Interestingly, Hnf1α is required for proper β-cell function and mutations in this gene cause MODY [23]. miR-192 is also expressed in the liver and is in cluster with miR-194, suggesting co-regulation [49,50]. Furthermore, miR-128 has been shown to induce apoptosis in kidney cells through interaction with Bax [51]. So far, miR-672 and miR-708 have not been examined in β-cells. The likely involvement of miR-128/192/194/204/708 in β-cell regulatory networks (Figure 5) and the high expression in β-cells compared to α-cells for miR-672/204 (Additional file 7) make them interesting candidates for further studies.

We have used ICA for bioinformatics investigation of the functional roles of the miRNAs and their targets. ICA in combination with pathway analysis indicates that the eight miRNAs, through their mRNA targets, are implicated in several diabetes relevant pathways.

The transcriptional changes mediated by miRNAs on their targets may not be entirely explained by direct repression but may also reflect indirect mechanisms such as activation by feedback and feed-forward transcriptional loops within regulatory networks [52,53]. miRNAs can be important players in these networks. By use of a combined bioinformatics approach we identified miRNA regulatory networks. The results suggest connections between seven of the eight miRNAs through interactions with key pancreatic transcription factors, cytokine signalling molecules and insulin (Figure 5).

## Conclusions

Using ICA, we have developed a novel miRNA target prediction method that incorporates seed matching and expression profiling. We believe that the method has advantages compared to simple negative correlation. Additionally, ICA in combination with bioinformatics approaches such as pathway analysis constitutes a means of prioritizing between the predicted miRNA targets for further investigations. To the best of our knowledge this is the first study that uses ICA for miRNA target prediction. Interestingly, applying the method on a model of type 1 diabetes resulted in identification of eight miRNAs that appear to directly or indirectly affect pathways of relevance to disease mechanisms in diabetes.

## Methods

### Cell cultures

INSrαβ, βTC-3 and αTC-1 cells were cultured in complete RPMI-1640 medium (RPMI-1640 with Glutamax (Gibco BRL, Paisley, Scotland, UK), 100 U/ml penicillin and 100 μg/ml streptomycin) supplemented with 10% FBS. 50 μM β-mercaptoethanol was additionally added to the INSrαβ cells. For the INSrαβ cells, TeT system approved FBS (CLONTECH, Palo Alto, CA) was used, and the medium was supplemented with 150 μg/ml geneticin and 100 μg/ml hygromycin to maintain a pure culture of cells expressing the Pdx-1 plasmid construct. The INSrαβ cells were treated with doxycycline (500 ng/ml; Sigma Aldrich, Saint Louis, MO, USA) for 24 h to allow for Pdx-1 expression after which the cells were cultured in the presence or absence of human recombinant IL-1β (40 pg/ml; BD Pharmingen, San Diego, CA) for additionally 2 h or 24 h.

### Human pancreatic islet preparations

Human islet preparations (n = 4) provided through the Juvenile Diabetes Research Foundation (JDRF) Islet Distribution Program (JDRF award 6-2005-1178) by Islet Cell Resource Centres in Milan (Italy) and Lille (France) were treated for 48 hours with IL-1β (1 ng/ul) or a combination of IL-1β (1 ng/ul), IFNγ (20 ng/ul) and TNFα (8 ng/ul).

### RNA extraction and RT-qPCR

Total RNA was extracted using TRIzol reagent (Invitrogen, Carlsbad, CA) according to the manufacturer's instructions. cDNA was prepared using miScript reverse transcription kit (Qiagen, Hilden, Germany), TaqMan microRNA reverse transcription reagents (Applied Biosystems, Foster City, CA) or TaqMan reverse transcription reagents (Applied Biosystems), as described by the manufacturer.

In INSrαβ cells (n = 5) the miRNA expression levels were analysed by use of miScript Primer Assays (Qiagen), whereas in βTC-3 (n = 5) and αTC-1 (n = 5) cells miRNA expressions were analysed using TaqMan microRNA assays (Applied Biosystems). Gene expression levels were analysed using TaqMan gene expression assays (Applied Biosystems). All samples were analyzed in duplicates on a ABI 7900HT system (Applied Biosystems). Data were evaluated using the 2^-ΔΔCt ^method [54], normalizing miRNA/gene expressions to an endogenous control, and subsequently comparing each miRNA/gene in treated vs. un-stimulated cells. miRNAs were normalized to let-7c, whereas genes were normalized to Ppia. The coefficient of variation (CV) for miScript assays ranged from 0.018 to 0.077.

### Western blotting

The INSrαβ cells (n = 4) were washed with PBS and lysed. The protein concentrations of whole cell protein lysates were measured with Bio-Rad Protein Assay. Samples were boiled before loading on a NuPage gel (10% BisTris gels, Invitrogen) placed in an X-Cell Surelock system (Invitrogen). Proteins were separated and then transferred to a pre-soaked nitrocellulose membrane (Invitrogen), which was blocked in milk for one hour and then washed. The membrane was incubated in a 5 ml milk solution containing primary antibody, either monoclonal Pdx-1 mouse antibody [37] overnight or monoclonal mouse β-actin antibody (Abcam, Cambridge, MA; #ab6276) for one hour. Horseradish peroxidase conjugated anti-mouse antibodies (Cell signalling, #7076) were used as secondary antibodies. The membrane was incubated in LumiGlo (Cell Signialing Technology, Danvers, MA) solution and visualized using the LAS2000 system (Fujifilm Europa GmbH, Dusseldorf, Germany).

### Profiling and preprocessing of array data

The mRNA expressions from INSrαβ cells (n = 4) were profiled on Affymetrix Rat Genome 230 2.0 arrays. For each probeset in each sample, log2-ratios were calculated between the expression of that probeset and the average of the control samples.

The miRNA expressions from INSrαβ cells (n = 3) were profiled on Exiqon miRCURY LNA microRNA Array v.11.0 arrays and median normalized. The control samples (n = 3) were pooled and used as a common reference on all arrays, and log2-ratios were calculated for each sample against this reference.

The mRNA expressions from human islet preparations (n = 4) were analyzed on Affymetrix Human Genome U133 Plus 2.0 arrays. The β-cell data from Refs. [41] and [42] were downloaded from T1DBase http://www.t1dbase.org. These mRNA expressions had been profiled using the Affymetrix Rat Genome U34A array.

All CEL-files were preprocessed using the RMA package [55] in Bioconductor [56] with remapped Ensembl build 50 gene probesets [57].

For mRNA data from human islets and published β-cell studies, foldchanges log2(cytokine, 24 h)/(untreated, 24 h) were calculated for the average of the replicate samples in each condition.

### Sequence based miRNA target prediction

For 7764 out of the 9953 Ensembl gene probesets analyzed, 3'UTR sequences could be downloaded from Ensembl through Biomart [58]. Mature sequences of significantly changed miRNAs were downloaded from miRbase [59] and the 6mer seed matches extracted. Ensembl gene probesets with a given miRNA 6mer seed match in their 3'UTR were identified as targets of that miRNA.

### Independent component analysis

Mathematically, *T *transcriptional programs are active in the cells under study, each of which is a (column) vector ${\vec{C}}_{t}$, *t *= 1, ..., *T *, representing *G*_*t *_gene inductions or repressions. We measure all *G *induced or repressed genes in *S *samples.

Each of these programs can be identified by a simple linear decomposition of the *G *× *S *expression matrix E,

$$\text{E}={\text{CM or E}}_{\text{gs}}=\sum_{t=1}^{T}C_{gt}M_{ts}\text{ or }{\vec{E}}_{s}=\sum_{t=1}^{T}C_{t}M_{ts}$$

where *C *is the *G *× *T *matrix containing the transcriptional programs, in coefficient vector notation, $C=\left({\vec{C}}_{1},{\vec{C}}_{2},\ldots ,{\vec{C}}_{T}\right)$, likewise $E=\left({\vec{E}}_{1},{\vec{E}}_{2},\ldots ,{\vec{E}}_{S}\right)$ and *M *is the *T *× *S *matrix giving the linear mixing of each program in each sample. Two-way clustering of correlated genes and samples into non-overlapping sets was represented by component vectors ${\vec{C}}_{t}$ with discrepant sets of non-vanishing entries

$$C_{gt}\neq 0\Leftrightarrow C_{gj}\approx 0\;\forall \;j\neq t.$$

ICA extracts n = min{*G, S*} independent components. Since the number of samples was much lower than the number of genes, we assumed n = *S*. The number of interesting transcriptional programs *T *may be equal to, or lower than *S*. A common practice is to project E onto it's first *O *principal component directions, and then extract *O *independent components in this subspace. This approach has several important drawbacks. First, the number of interesting transcriptional programs is not known beforehand, and one therefore needs to guess or resort to a recursive trial-and-error approach to choose an *O *that allows all *T *transcriptional programs to be extracted. Second, it is not obvious that a projection onto *O *<*S *principal component directions preserves all *T *transcriptional programs. It is possible to construct examples where two clusters in the data are only well separated in the subspace of the first and last principal components [60]. Clustering in a subspace spanned by principal components generally degrades cluster quality [61]. Finally, even when a projection onto *T *principal components allows all the *T *interesting transcriptional programs to be extracted by ICA, a way to explore and rank these independent components to clarify their significance is still needed. In the present study, the independent components were ranked according to their accordance with the experimental conditions.

The fastICA algorithm [62,63] in R http://www.R-project.org was used to estimate the component matrix and mixing matrix, by numeric maximization of the negentropy of the independent components. The negentropy was approximated by J(y) = (〈G(y)〉 - 〈G(v)〉)^2^, where y is the distribution of loads, v is a random variable distributed as ℵ(0,1), and the contrast function G(u) was here defined as $\text{G(u)}=\text{-exp}\left(\frac{{\text{-u}}^{\text{2}}}{2}\right)$. By convention, the loads in each component have mean 0 and variance 1.

Assuming that the individual contributions on mRNA expression are linearly separable and reasonable independent of each other, ICA can separate the various contributions making it possible to detect pathways regulated by the various miRNAs.

ICs with mixes, i.e. rows in *M *showing significant changes between the experimental conditions and/or time points, are identified by calculating F-statistics and requiring Bonferroni corrected p-values less than 0.0001.

### Statistics

Overall expression data were analysed using one-way analysis of variance (ANOVA; F-statistics). The eight significant miRNAs were selected based on an overall q-value < 0.1 (Additional file 8). Comparison between groups was done using t-test. Correction for multiple testing was performed either by applying a Bonferroni or a false discovery rate (fdr) correction to the p-values (q-value). A Bonferroni correction was chosen when few tests were performed, as was the case for the finding of significant ICs and miRNAs. When a larger number of tests were performed a fdr correction was applied.

The loads in each IC are considered non-Gaussian distributed. Loads/fold changes for genes that had seed match for the miRNAs or were assigned to a specific biological pathway were analysed using Wilcoxon rank sum test. For analysis of negative correlation, Pearson correlation coefficients were calculated between each pair of miRNA and mRNA profiles. The mRNAs were then ordered according to these correlation coefficients. P-values < 0.05 were considered significant.

## List of abbreviations

**3'UTR**: untranslated region in the 3' end of the mRNA; **dox**: doxycycline; **FBS**: fetal bovine serum; **IC**: independent component; **ICA**: independent component analysis; **IFNγ**: interferon gamma; **IL-1β**: interleukin 1 beta; **miR/miRNA**: microRNA; **MODY**: maturity onset diabetes of the young; **mRNA**: messenger RNA; **PBS**: phosphate buffered saline; **PCA**: principal component analysis; **RT-qPCR**: reverse transcription - quantitative polymerase chain reaction; **T1D**: type 1 diabetes; **T2D**: type 2 diabetes; **TNFα**: tumor necrosis factor alpha.

## Authors' contributions

FP and CHBB designed the study. The experimental work was performed by CHBB, TF and TG. The analyses and the manuscript draft were conducted by LP, CHBB and TF. ICA and microarray data analysis were conducted by LP and PHH. FP edited the paper and prepared it for final review. All authors read and approved the final manuscript and have no conflicting interest in the study.

## Supplementary Material

Additional file 1 **Expressions of insulin and Pdx-1 dependent genes**. There are three experimental conditions: Pdx-1 induction (dox treatment), IL-1β treatment and time (samples are taken 2 h and 24 h after treatment). Log2-transformed fold changes (mean and standard deviation) between experimental and control conditions. *: 0.05 > q > 0.01, **: 0.01 > q > 0.001, ***: 0.001 > q > 0. Click here for file

Additional file 2 **miRNA target prediction based on mRNA expression data**. For each Ensembl annotated gene on the Affymetrix array is given the gene symbol, gene description, loads in the five ICs, coefficients for correlation with the eight miRNA expression profiles and 6mer seed match with the eight miRNAs. We assumed that loads were significant if they had an absolute load greater than 2. For seed match, 1/0 denotes ≥1 seed matches and no seed match. Click here for file

Additional file 3 **Cooperativity between miRNAs in IC 1**. The p-values (uncorrected) and q-values (corrected) for pairwise miRNA cooperativity in IC 1 are shown. 1/-1 denotes whether the individual miRNA has positive or negative loads in IC 1. Click here for file

Additional file 4 **Coefficients for the linear superposition of the ICs giving the miRNA expression profiles**. R2 is the coefficient of determination. Click here for file

Additional file 5 **Genes regulated by Pdx-1 and/or IL-1β**. 1/0 denotes regulation and no regulation based on text mining. Click here for file

Additional file 6 **Pathways significantly enriched for genes with high positive or low negative loads in the ICs**. Sheet 1 shows KEGG pathways whereas sheet 2 shows MSigDB pathways. Only pathways with q-values < 0.05 (fdr corrected p-values) are highlighted. Click here for file

Additional file 7 **Expression of the eight miRNAs in α- versus β-cells**. Ratio of basal ΔCT values for αTC1 versus βTC3 cells. The ratio is found for un-stimulated cells. Bars are standard deviations and asterisks denote: **: 0.01 > q > 0.001, ***: 0.001 > q > 0. Click here for file

Additional file 8 **Overall q-values for the miRNA expressions**. FWER: family-wise error rate. Click here for file
