# Supplementary figures and images for: Independent component and pathway-based analysis of miRNA-regulated gene expression in a model of type 1 diabetes
Source: BMC Genomics. 2011 Feb 4;12:97. doi: 10.1186/1471-2164-12-97 (PMC3040732; doi:10.1186/1471-2164-12-97)

log2(fold change)

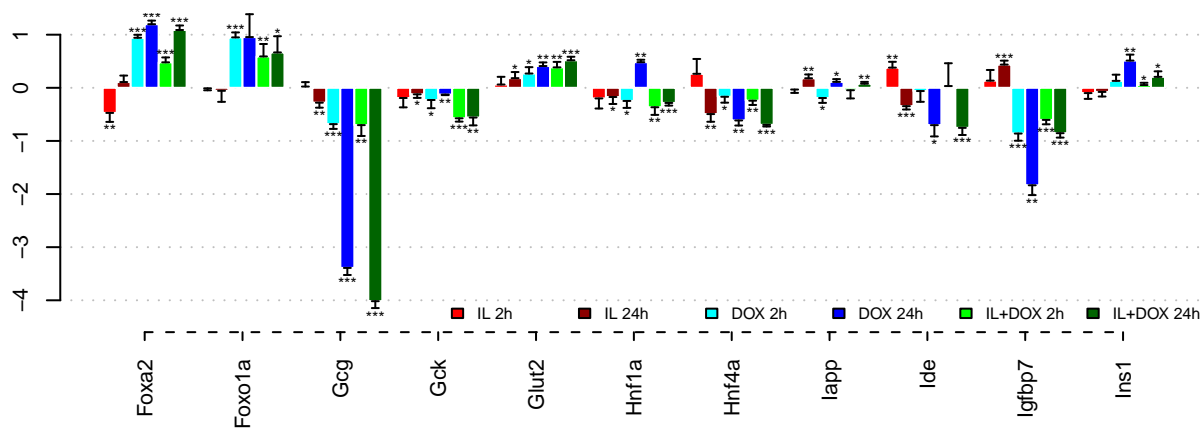

log2(fold change)

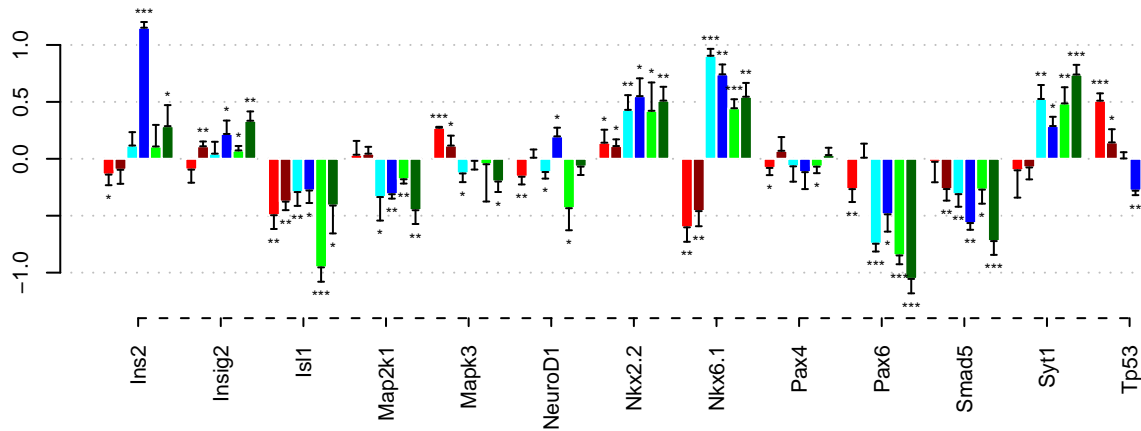

Supplement: Additional file 1 — Expressions of insulin and Pdx-1 dependent genes. There are three experimental conditions: Pdx-1 induction (dox treatment), IL-1β treatment and time (samples are taken 2 h and 24 h after treatment). Log2-transformed fold changes (mean and standard deviation) between experimental and control conditions. *: 0.05 > q > 0.01, **: 0.01 > q > 0.001, ***: 0.001 > q > 0. [file 1471-2164-12-97-S1.PDF]

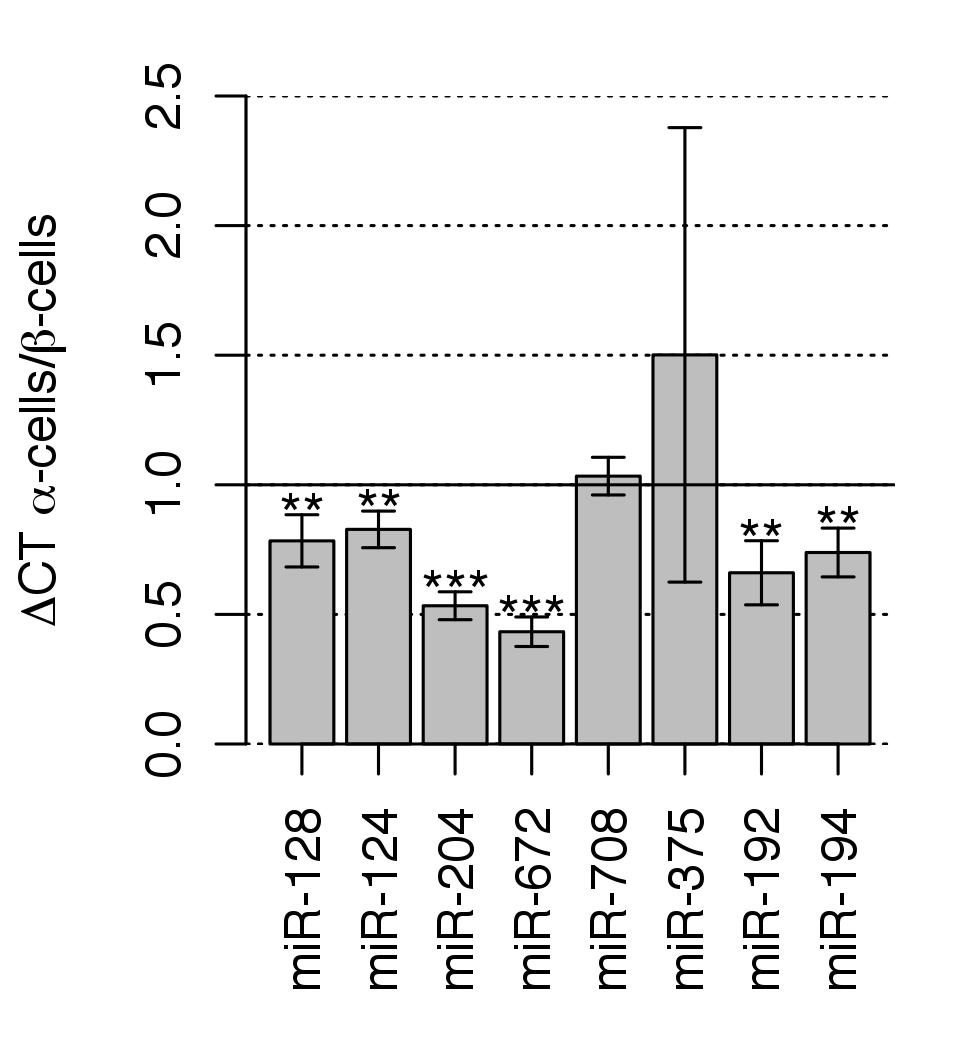

Supplement: Additional file 7 — Expression of the eight miRNAs in α- versus β-cells. Ratio of basal ΔCT values for αTC1 versus βTC3 cells. The ratio is found for un-stimulated cells. Bars are standard deviations and asterisks denote: **: 0.01 > q > 0.001, ***: 0.001 > q > 0. [file 1471-2164-12-97-S7.JPEG]
